# Fitting a shared frailty illness-death model to left-truncated semi-competing risks data to examine the impact of education level on incident dementia

**DOI:** 10.1186/s12874-020-01203-8

**Published:** 2021-01-11

**Authors:** Catherine Lee, Paola Gilsanz, Sebastien Haneuse

**Affiliations:** 1 Kaiser Permanente Northern California, Division of Reseach, 2000 Broadway, Oakland, CA US; 2 Harvard T.H. Chan School of Public Health, 677 Huntington Avenue, Boston, MA US

**Keywords:** Illness-death, Left-truncation, Semi-competing risks, Multistate models, B-splines, Dementia

## Abstract

**Background:**

Semi-competing risks arise when interest lies in the time-to-event for some non-terminal event, the observation of which is subject to some terminal event. One approach to assessing the impact of covariates on semi-competing risks data is through the illness-death model with shared frailty, where hazard regression models are used to model the effect of covariates on the endpoints. The shared frailty term, which can be viewed as an individual-specific random effect, acknowledges dependence between the events that is not accounted for by covariates. Although methods exist for fitting such a model to right-censored semi-competing risks data, there is currently a gap in the literature for fitting such models when a flexible baseline hazard specification is desired and the data are left-truncated, for example when time is on the age scale. We provide a modeling framework and openly available code for implementation.

**Methods:**

We specified the model and the likelihood function that accounts for left-truncated data, and provided an approach to estimation and inference via maximum likelihood. Our model was fully parametric, specifying baseline hazards via Weibull or B-splines. Using simulated data we examined the operating characteristics of the implementation in terms of bias and coverage. We applied our methods to a dataset of 33,117 Kaiser Permanente Northern California members aged 65 or older examining the relationship between educational level (categorized as: high school or less; trade school, some college or college graduate; post-graduate) and incident dementia and death.

**Results:**

A simulation study showed that our implementation provided regression parameter estimates with negligible bias and good coverage. In our data application, we found higher levels of education are associated with a lower risk of incident dementia, after adjusting for sex and race/ethnicity.

**Conclusions:**

As illustrated by our analysis of Kaiser data, our proposed modeling framework allows the analyst to assess the impact of covariates on semi-competing risks data, such as incident dementia and death, while accounting for dependence between the outcomes when data are left-truncated, as is common in studies of aging and dementia.

**Supplementary Information:**

The online version contains supplementary material available at (10.1186/s12874-020-01203-8).

## Background

Semi-competing risks refers to the setting where interest lies in the time-to-event for some so-called *non-terminal* event, the observation of which is subject to some *terminal* event [1]. In contrast to standard competing risks, where each of the outcomes under consideration is typically terminal (e.g. death due to some cause or another), in the semi-competing risks setting it is possible to observe both events on the same study unit, so that there is at least partial information on their joint distribution [1, 2]. Take as an example the study of dementia among the elderly [3], a complex neurocognitive condition that is estimated to affect nearly 6 million individuals aged 65 and older in the US [4], a number that has been projected increase to 13.9 million by 2060 [5]. It is known that the risk of death is higher among those who are diagnosed with dementia [6]. As such, studies seeking to investigate risk factors for dementia must contend with death as a competing risk, which precludes the subsequent observation of dementia. However, it is possible to observe both outcomes among individuals who die following a diagnosis of dementia. This information can potentially increase efficiency of results and be used to assess the dependence between the nonterminal and terminal events.

Towards the analysis of semi-competing risks data, the statistical literature has focused on three broad frameworks that seek to exploit the joint information on the time to the non-terminal event and the time to the terminal event [7]: those based on copulas [1, 8–10]; those framed from the perspective of causal inference [11, 12]; and, those based on the illness-death multi-state model [2, 13–16]. In this paper, we focus on the last of these approaches, for which the philosophical underpinning is that patients begin in some initial state at time zero and may transition into the non-terminal and/or terminal state [14, 16–18]. Analyses typically proceed through the development of models for transition-specific hazard functions (which dictate the rate at which patients experience the events), often with the use of subject-specific frailties, which can be viewed as individual-specific random effects that acknowledge the heterogeneity across individuals that is not accounted for by covariates [19, 20]. Moreover, the shared frailty accounts for dependence between the nonterminal and terminal events, which can be quantified from an estimable frailty variance parameter.

In the analysis of time-to-event outcomes, data are subject to left-truncation or delayed entry when subjects are enrolled into a study after the time origin of interest. Left-truncation is common in the study of aging and dementia, where the age scale is commonly taken to be the time scale [21–23]. In this setting, sampling is biased toward longer follow-up times since patients are typically only included in the study if they are dementia-free at study entry. The analysis of left-truncated time-to-event data should apply statistical methods that account for this bias. Although current methods exist for analyzing left-truncated semi-competing risks data via a standard illness-death model (without a shared frailty) [24, 25] and have been applied to Alzheimer’s disease [26–29], to our knowledge, there are no published methods in the literature for fitting an illness-death model with shared frailty to left-truncated semi-competing risks data. The purpose of this paper is to fill this gap by providing a modeling framework and openly available code for implementation.

In this paper, we provide methods for fitting an illness-death model with shared frailty to left-truncated semi-competing risks data. In “Methods” section, we present the model specification (“Model specification: Illness-death model” section), methods for estimation and inference (“Estimation and inference” section), a brief simulation study (“Simulation study” section), and an analysis of data from Kaiser Permanente Northern California examining the relationship between educational level and incident dementia and death (“Assessing the impact of education level on incident dementia in a large US cohort” section). We present results in “Results” section. We conclude with a discussion in “Discussion” section and conclusions in “Conclusions” section.

## Methods

### Model specification: Illness-death model

**Fig. 1:**
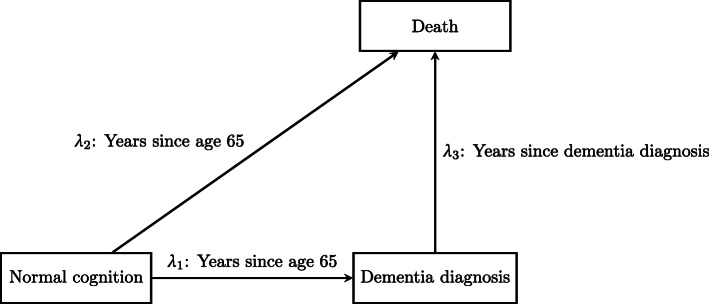
Schematic of an illness-death model

Semi-competing risks refers to the setting where interest lies in a nonterminal event that is subject to dependent censoring by a terminal event [1]. This paper focuses on modeling semi-competing risks data using the illness-death multistate model, where in the study of dementia we take the nonterminal and terminal events to be dementia diagnosis and death. Figure 1 illustrates the possible individual trajectories following time of study accrual. Let *T*_1_ and *T*_2_ denote the nonterminal and terminal event times, respectively, following a well-defined time origin. Following the multistate modeling literature, the illness-death model is characterized by the transition hazards:

1 $$\begin{array}{*{20}l} \lambda_{1}(t_{1})\ &={\lim}_{\Delta\to 0} P(T_{1}\in [t_{1},t_{1}+\Delta)|T_{1} \end{array} $$

2 $$\begin{array}{*{20}l} &\ge t_{1}, T_{2}\ge t_{1})/\Delta, ~\text{for }t_{1}>0 \\ \lambda_{2}(t_{2})\ &={\lim}_{\Delta\to 0} P(T_{2}\in [t_{2},t_{2}+\Delta)|T_{1} \end{array} $$

3 $$\begin{array}{*{20}l} &\ge t_{2}, T_{2}\ge t_{2})/\Delta,~\text{for }t_{2}>0 \\ \lambda_{3}(t_{2}|T_{1}=t_{1})\ &=&{\lim}_{\Delta\to 0} P(T_{2}\in [t_{2},t_{2}+\Delta)|T_{1} \\ &=t_{1}, T_{2}\ge t_{2})/\Delta,~\text{for }0< t_{1}< t_{2}. \end{array} $$

Although a variety of hazard regression models can be adopted, including Cox-type multiplicative hazard regression models [30], additive models [31] and accelerated failure time models [32], this paper assumes the first of these which is prevalent in the multistate modeling literature:

4 $$\begin{array}{*{20}l} \lambda_{1}(t_{1}| \mathbf{X}_{1})\ & =\ \gamma \cdot \lambda_{1,0}(t_{1}) \exp\{\beta_{1}\mathbf{X}_{1}\}, \end{array} $$

5 $$\begin{array}{*{20}l} &\text{for}\ t_{1} >0  \\ \lambda_{2}(t_{2}| \mathbf{X}_{2})\ & =\ \gamma \cdot \lambda_{2,0}(t_{2})\exp\{\beta_{2}\mathbf{X}_{2}\}, \end{array} $$

6 $$\begin{array}{*{20}l} & \text{for}\ t_{2}>0 \\ \lambda_{3}(t_{2}|T_{1}\,=\,t_{1},\mathbf{X}_{3})\ & =\ \gamma \cdot \lambda_{3,0}(t_{2}|T_{1}\,=\,t_{1})\exp\{\beta_{3}\mathbf{X}_{3}\},\\ &\text{for }0< t_{1}< t_{2}, \end{array} $$

where *λ*_*k*,0_(·) are baseline hazard functions and *β*_*k*_ are log-hazard ratio regression parameters, for *k*=1,2,3. We allow for two common specifications of *λ*_3,0_(*t*_2_|*T*_1_=*t*_1_), a Markov model defined by *λ*_3,0_(*t*_2_|*T*_1_=*t*_1_)=*λ*_3,0_(*t*_2_) or a semi-Markov (‘clock-reset’) model defined by *λ*_3,0_(*t*_2_|*T*_1_=*t*_1_)=*λ*_3,0_(*t*_2_−*t*_1_). In each of expressions (4)-(6), *γ* is a common subject-specific frailty with mean 1.0 and variance *θ*, which serves two related purposes. First, similar to a random intercept in a generalized linear mixed model [33], the frailties serve to accommodate between-subject heterogeneity that is not accounted for by the covariates included in the linear predictors. Second, the frailty induces dependence between the non-terminal and terminal events since a patient with frailty larger than one will be at higher risk of both the nonterminal and terminal events than the population average (conditional on covariates).

To complete the specification of the model, the baseline hazard functions and frailty distribution must be specified. For the latter, we adopt a Gamma distribution, which is commonly used because closed-form expressions for the marginal likelihood contributions can be obtained. It is important to note that although the shared frailty term can only account for or represent positive dependence between the hazard functions [20], it is possible to have negative correlation between the hazard functions corresponding to *T*_1_ and *T*_2_, e.g. if *λ*_1,0_(*t*) is monotonically increasing and *λ*_2,0_(*t*) is monotonically decreasing.

The baseline hazard functions can be specified parametrically [34], semi-parametrically [15, 26], or non-parametrically [2]. In this paper, we consider Weibull baseline hazards of the form $\phantom {\dot {i}\!}\lambda _{k,0}(t)=\alpha _{k} \kappa _{k} t^{\alpha _{k} - 1}$, for *k*=1,2,3, which are commonly used in survival analysis, and flexible B-spline baseline hazard functions that satisfy log*λ*(*t*)=*B*(*t*), where *B*(*t*) is a polynomial B-spline function of degree *d* with unique knots at $t_{0}< t_{1}< \dots < t_{K}< t_{K+1}$ and defined for *t*∈[*t*_0_,*t*_*K*+1_]. For continuous time-to-event outcomes with support on the positive real line, we let *t*_0_=0 and *t*_*K*+1_ to be the largest follow-up time. Note that the B-spline function *B*(*t*) is parametrically defined as a linear combination of B-spline basis functions *B*_*b*,*d*_(*t*) of degree *d*,

$$B(t)=\sum_{b=0}^{K+d} \eta_{b} B_{b,d}(t),$$

where *η*_*b*_ are parameters, known as control points, and the B-spline basis functions are defined for *t*∈[*t*_0_,*t*_*K*+1_] [35].

### Estimation and inference

#### Observed data

Let *T*_*i*1_ and *T*_*i*2_ denote the nonterminal and terminal event times and **X**_*i*_ a vector of patient-specific covariates that includes **X**_*i*1_,**X**_*i*2_ and **X**_*i*3_ for individual *i* used in (4)-(6). We assume that the observed data are subject to right-censoring and left-truncation. Let *C*_*i*_ and *L*_*i*_ denote the right-censoring and left-truncation times which we assume are independent of *T*_*i*1_ and *T*_*i*2_ conditional on **X**_*i*_. Note that we assume the convention of Xu and colleagues [2], by setting *T*_1_=*∞* for individuals who experience the terminal event in the absence of the nonterminal event. In the analysis of dementia on the age scale, where the nonterminal and terminal events are dementia diagnosis and death and the left-truncation time is study entry, prevalent dementia cases are typically excluded from the analysis since the primary outcome of interest is dementia [27], i.e. we require that *T*_*i*1_>*L*_*i*_. The observed data for the *i*^*th*^ individual is *D*_*i*_={*L*_*i*_,*Y*_*i*1_,*δ*_*i*1_,*Y*_*i*2_,*δ*_*i*2_,**X**_*i*_}, where *Y*_*i*1_= min(*T*_*i*1_,*T*_*i*2_,*C*_*i*_) with nonterminal event indicator *δ*_*i*1_=*I*{*T*_*i*1_≤ min(*T*_*i*2_,*C*_*i*_)},*Y*_*i*2_= min(*T*_*i*2_,*C*_*i*_) with terminal event indicator *δ*_*i*2_=*I*{*T*_*i*2_≤*C*_*i*_}, and *L*_*i*_<*Y*_*i*1_.

#### Likelihood

Towards developing a form of the likelihood, we first present the joint density, (*T*_1_,*T*_2_), in the absence of left-truncation below [15, 25]:

7 $$\begin{array}{@{}rcl@{}} \lefteqn{g_{T_{1},T_{2}}(t_{1},t_{1}|X) } \\ &=& \left\{\begin{aligned} &\lambda_{1}(t_{1}|X)\lambda_{3}(t_{2}|t_{1},X)S_{1}(t_{1}|X)S_{2}(t_{1}|X)S_{3}(t_{2}|t_{1},X),\\&\text{for}~0< t_{1}\le t_{2}\\ &\lambda_{2}(t_{2}|X)S_{1}(t_{2}|X)S_{2}(t_{2}|X),\\&\text{for}~0< t_{2}\le t_{1} = \infty, \\ \end{aligned} \right. \end{array} $$

where $S_{k}(t|X)=\exp \left \{-\int _{0}^{t} \lambda _{k}(u|X)~du\right \}$, for *k*=1,2, and either $S_{3}(t_{2}|t_{1},X)=\exp \left \{-\int _{t_{1}}^{t_{2}} \lambda _{3}(u|t_{1},X)~du\right \}$ if a Markov model is assumed for *λ*_3_(·) or $S_{3}(t_{2}|t_{1},X)=\exp \left \{-\int _{0}^{t_{2}-t_{1}} \lambda _{3}(u|t_{1},X)~du\right \}$ if a semi-Markov model is assumed.

Under the assumption of independent truncation, the joint density of left-truncated semi-competing risks data, (*L*,*T*_1_,*T*_2_), is:

8 $$\begin{array}{*{20}l} f_{T_{1},T_{2},L}(t_{1},t_{2},l|X) &=f_{T_{1},T_{2},L}(t_{1},t_{2},l|L<T_{1},X) \\ &=\frac{g_{T_{1},T_{2}}(t_{1},t_{2}|X)g_{L}(l)}{P(L<T_{1}|X)}I(l< t_{1})\\ &=\frac{g_{T_{1},T_{2}}(t_{1},t_{2}|X)g_{L}(l)}{\int S_{T_{1}}(l|X)g_{L}(l)~dl}I(l< t_{1}) \\ &={\frac{g_{T_{1},T_{2}}(t_{1},t_{2}|X)I(l< t_{1})}{S_{T_{1}}(l|X)}}\\&\cdot {\frac{S_{T_{1}}(l|X) g_{L}(l)}{\int S_{T_{1}}(l|X)g_{L}(l)~dl}},  \end{array} $$

which is a product of the conditional density of (*T*_1_,*T*_2_) given *L* and the marginal density of *L* [24, 25, 36] in (8), where $g_{T_{1},T_{2}}$ is given in (7), *g*_*L*_(·) is the density function corresponding to *L* and $S_{T_{1}}(t|X)=P(T_{1}>t|X)=S_{1}(t|X)S_{2}(t|X)$. When the distribution of left-truncation times is unknown, the maximum likelihood estimate can be obtained from the conditional likelihood, ignoring the marginal likelihood of the left-truncation times [24, 25, 36, 37].

Based on the observed data, *D*_*i*_, there are four possible data scenarios and thus likelihood contributions corresponding to the distinct combinations of the nonterminal and terminal event indicators, *δ*_1_ and *δ*_2_. Using (7) and the conditional likelihood expression (on the left) in (8), the observed data likelihood for model parameters *ϕ*=(*ξ*_1_,*ξ*_2_,*ξ*_3_,*β*_1_,*β*_2_,*β*_3_,*θ*), where *ξ*_*k*_ are the baseline hazard parameters and *β*_*k*_ are the vectors of regression coefficients for transition *k*=1,2,3 and *θ* is the Gamma frailty variance, is

$$\begin{array}{@{}rcl@{}} \lefteqn{ \mathcal{L}(\phi)} \\ &=&\ \prod_{i=1}^{n} \left[ f_{1}(Y_{i1},Y_{i2}|X_{i})^{\delta_{i1}\delta_{i2}} \cdot f_{2}(Y_{i1},Y_{i2}|X_{i})^{\delta_{i1}(1-\delta_{i2})} \right.\\ & & \left.\cdot f_{3}(Y_{i1},Y_{i2}|X_{i})^{(1-\delta_{i1})\delta_{i2}} \cdot f_{4}(Y_{i1},Y_{i2}|X_{i})^{(1-\delta_{i1})(1-\delta_{i2})} \right] \end{array} $$

where

9 $$\begin{array}{@{}rcl@{}} f_{k}(Y_{i1}, Y_{i2}|X_{i}) &=& \int f_{k}(Y_{i1}, Y_{i2}|X_{i},\gamma_{i})f(\gamma_{i})~\partial\gamma_{i},~\\&&\text{for}~k=1,\dots,4,  \end{array} $$

and

$${}\begin{aligned} f_{1}(Y_{i1}, Y_{i2}|X_{i},\gamma_{i})&=\lambda_{1}(Y_{i1}|X_{i},\gamma_{i})\lambda_{3}(Y_{i2}|X_{i},\gamma_{i})S_{1}(Y_{i1}|X_{i},\gamma_{i})\\&S_{2}(Y_{i1}|X_{i},\gamma_{i})S_{3}(Y_{i2}|Y_{i1},X_{i},\gamma_{i})/\\ &[S_{1}(L_{i}|X_{i},\gamma_{i})S_{2}(L_{i}|X_{i},\gamma_{i})]\\ f_{2}(Y_{i1}, Y_{i2}|X_{i},\gamma_{i})&=\lambda_{1}(Y_{i1}|X_{i},\gamma_{i})S_{1}(Y_{i1}|X_{i},\gamma_{i})\\&S_{2}(Y_{i1}|X_{i},\gamma_{i})S_{3}(Y_{i2}|Y_{i1},X_{i},\gamma_{i})/\\ &[S_{1}(L_{i}|X_{i},\gamma_{i})S_{2}(L_{i}|X_{i},\gamma_{i})]\\ f_{3}(Y_{i1}, Y_{i2}|X_{i},\gamma_{i})&= \lambda_{2}(Y_{i2}|X_{i},\gamma_{i})S_{1}(Y_{i2}|X_{i},\gamma_{i})\\&S_{2}(Y_{i2}|X_{i},\gamma_{i})/[S_{1}(L_{i}|X_{i},\gamma_{i})S_{2}(L_{i}|X_{i},\gamma_{i})]\\ f_{4}(Y_{i1}, Y_{i2}|X_{i},\gamma_{i})&=S_{1}(Y_{i1}|X_{i},\gamma_{i})S_{2}(Y_{i1}|X_{i},\gamma_{i})/\\&[S_{1}(L_{i}|X_{i},\gamma_{i})S_{2}(L_{i}|X_{i},\gamma_{i})] \end{aligned} $$

Since the individual-specific frailty terms, *γ*_*i*_, are not observed, we marginalize the likelihood with respect to *γ*_*i*_. Detailed derivations of the marginal likelihood components in (9) are included in Section A of the Additional file 1.

#### Estimation and inference

We will use maximum likelihood for estimation and inference of model parameters using the marginal log-likelihood in Section A of the Additional file 1 [38]. Let $\mathcal {U}(\phi) = \partial /\partial \phi \left (\log \mathcal {L}(\phi)\right)$ denote the score function. Using standard arguments, under certain regularity conditions and a correctly specified model, the maximum likelihood estimator of *ϕ*, denoted $\widehat {\phi }$, is the solution to $\mathcal {U}(\phi) = 0$ and is consistent for the true *ϕ*_0_ as *n* →*∞*. In addition, $\sqrt {n}(\widehat {\phi } -\phi _{0}) \longrightarrow _{d}$ MVN (0, *Σ*) as *n* → *∞*, where *Σ*=*I*(*ϕ*_0_)^−1^ is the inverse of the expected information matrix:

$$\begin{array}{@{}rcl@{}} I(\phi_{0})\ &=&\ -E\left.\left[\frac{\partial^{2}}{\partial\phi^{2}}\log{L(\phi)} \right]\right|_{\phi=\phi_{0}}. \end{array} $$

Var[$\widehat {\phi }$] can be estimated via the inverse of the observed information matrix.

From our experience fitting the proposed model, optimization of the log-likelihood requires careful consideration of the numerical optimization algorithm used and the choice of starting values. For modeling fitting, we used a quasi-Newton non-linear numerical optimization algorithm [39] as implemented in the optim function in R [40] to maximize the log-likelihood. For Weibull baseline hazard models, starting values for the non-linear optimization were generated by fitting the univariate Weibull regression models for each transition (4)-(6). For B-spline parameterizations of the baseline hazard functions, we used the bSpline function in the splines2 package [41] in R [40] to generate B-spline basis functions and obtained starting values for hazard parameters as follows: fit univariate Cox models for each transition hazard model; smoothed the estimated cumulative baseline hazard functions using linear interpolation; obtained smoothed baseline hazards function via numerical differentiation followed by loess; and found the control points that minimized the distance between the smoothed log-hazard functions and B-spline functions by least squares. The frailty variance *θ* was initialized to value 0.5.

### Simulation study

We performed a set of simulations to investigate the operating characteristics (e.g., bias and coverage) of the model implementation for both Weibull and B-spline parameterizations of the baseline hazard functions.

Data were generated from the model (4)-(6) assuming Weibull baseline hazard functions using simID from the SemiCompRisks package [42, 43] in R [40]. We set model parameters to those obtained from fitting the model to dementia data, where the nonterminal and terminal events were dementia diagnosis and death, the origin was age 65, the left-truncation time was study entry, and a dichotomous variable was included as a covariate in all three regression models. The baseline hazard and frailty parameter values used in the simulations were: log*α*_1_=1.05, log*κ*_1_=−9.98, log*α*_2_=1.15, log*κ*_2_=−10.01, log*α*_3_=0.92, log*κ*_3_=−5.92, and log*θ*=−1.39. The dichotomous covariate was drawn from a Bernoulli distribution with probability 0.57 and the regression coefficients for the three transitions were *β*_1_=−0.03,*β*_2_=−0.33 and *β*_3_=−0.11. Age at study entry was drawn from a uniform distribution with range between 65 and 78 years. We administratively censored at age 95 years.

Based on these set parameters, we simulated *R*=1,000 datasets of sample size *n*=5,000. For each simulated dataset, we fit both the model with Weibull and B-spline baseline hazard functions. For the fitted Weibull models, we reported the: mean of the parameter estimates; mean of the estimated analytical standard errors; standard deviation of the distribution of parameter estimates (empirical standard error); and the coverage probability (the proportion of Wald-based 95% confidence intervals that contained the true parameter). For the fitted B-spline-based models, we reported the operating characteristics listed for the Weibull-based fitted models for the regression and frailty parameters only. To assess the B-spline fit of the transition baseline hazard functions, for each of the three transitions, we plotted the estimated baseline hazards from the fitted models, which we compared to the true baseline hazard. We also compared the estimated baseline hazard functions to kernel smoothed Nelson-Aalen baseline hazard estimates obtained from fitting separate Cox models to each transition using the coxphHaz function from the biostat3 package [44] in R [40]. Note that the univariate Cox models for the 1- and 2-transitions accounted for left-truncation and the Cox model on the 3-transition was performed only among those who experienced the nonterminal event. To quantify the difference between the true baseline hazard function and those estimated from the B-spline model and the smoothed Nelson-Aalen, we calculated the median of the integrated squared error [26].

### Assessing the impact of education level on incident dementia in a large US cohort

We applied the methodological approach presented in “Model specification: Illness-death model” and “Estimation and inference” sections to data from Kaiser Permanente Northern California (KPNC) where the goal of the analysis was to examine the association of education level on incident dementia. The initial analytic cohort consisted of 36,134 individuals who were at least 65 years old, dementia-free by 1997 and KPNC members as of January 1, 1996 (the earliest date when electronic medical records were reliably available defined as study entry) and participated in the Multiphasic Health Checkups which began in the 1960s and continued through 1996 during which information on participant educational attainment was captured. We categorized the exposure of educational attainment level based on the highest level of completion in the following groups: elementary or high school; trade degree, any college or college graduate; and post-graduate study. We focused the analyses on the four most prevalent race/ethnic subgroups, which were White, Black, Asian and Latinx, resulting in a final analytic dataset comprised of 33,117 KPNC members.

We incorporated the strong force of mortality (of all causes) of individuals aged 65 or greater in the analysis via the illness-death model in (1)-(3), where the nonterminal event of interest was incident dementia diagnosis and the terminal event was death. We assumed the semi-Markov or ‘clock-reset’ approach for the 3-transition model (dementia → death). Follow-up was administratively censored at the earliest of age 90 or end of study defined by September 30, 2017. In our primary analysis, we used a shared frailty to account for the dependence between incident dementia and death that is not accounted for by covariates. We assumed that time was on the age scale, starting at age 65, and thus left-truncated since members were not followed before age 65. For this time origin, left-truncation was accounted for using the approach in “Estimation and inference” section, assuming B-spline parameterizations of the transition baseline hazard functions. When B-spline baseline hazards are assumed, model fitting requires the specification of the internal knots (number and placement) and polynomial degree of the B-spline function. We fit a suite of models that varied the number of internal knots (we considered 1, 2 or 3 knots), which were placed at equally spaced percentiles of the observed event times, and varied the B-spline polynomial degree (linear, quadratic, cubic). The final model was chosen based on the largest log-likelihood. Time was scaled by a factor of five so that the interpretation of hazard ratios are in terms of 5-year increments. We controlled for sex (reference group: male) and race/ethnicity (reference group: White) in all hazard transition models via covariate adjustment.

In sensitivity analyses, we fit models: 1) without a shared frailty; 2) that defined study entry as the time origin (which we refer to as time-on-study models); and 3) assumed Weibull baseline hazard functions. When the origin was taken to be study entry, for the 1- and 2-transition hazard models we adjusted for age at study entry (centered at age 65); for the 3−transition hazard model, we adjusted for age at dementia (centered at the cohort mean which was 83 years). These models were fit using the SemiCompRisks package in R [42].

Note that this was an electronic medical record (EMR)-based study. KPNC is an integrated health care delivery system of approximately 4.3 million members that adopted an EMR system as early as 1993. All healthcare utilization, including care visits (inpatient, outpatient (ambulatory), emergency department, telephone and video visits), pharmacy fills, etc., are captured in the EMR. Dementia diagnoses and death dates were pulled from the EMR. Dementia diagnoses were based on International Classification of Diseases (ICD) codes that were made during a member visit. Deaths were obtained from the KPNC mortality linkage file which includes data from multiple sources (internal reporting, California state death records, Social Security Administration). Follow-up was not prescribed within the cohort and could vary by individual. However, health system utilization is very high in this population with a median of 97 visits with a physician during the study period (minimum: 0.003 years, median: 15.4 years, maximum: 21.2 years). Details regarding the dataset, including outcome definition, exposure variable and covariates, can be found in the Additional file 1: Section C.1.

## Results

### Simulation study

**Fig. 2:**
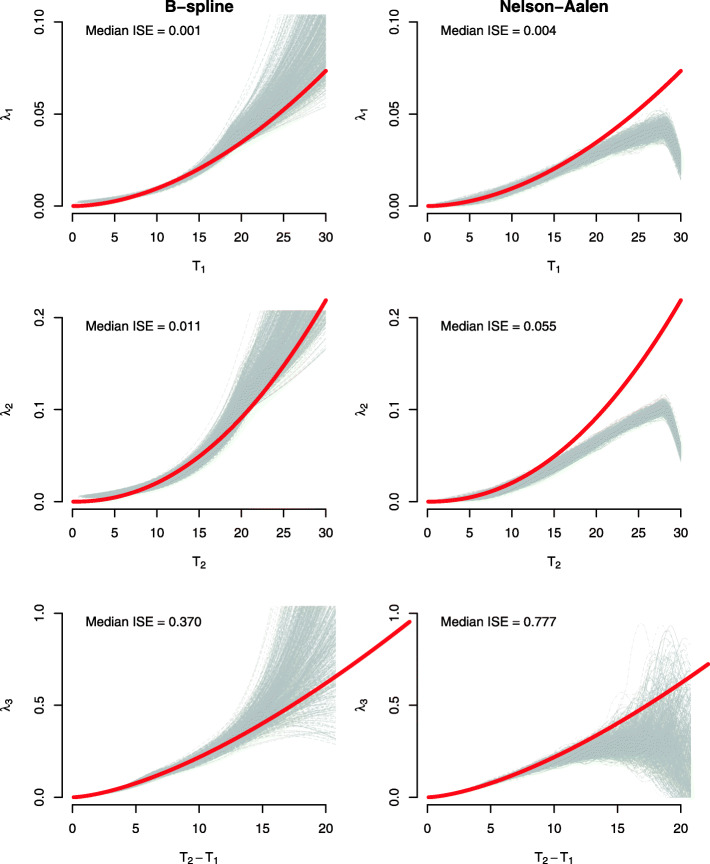
Simulation results corresponding to 1,000 simulated dataset. Presented are plots of true Weibull-based baseline hazards (red) for the three transition hazards compared to fitted baseline hazards from the B-spline parameterization and kernel smoothed Nelson-Aalen estimators on three separate Cox models. Median integrated squared error (ISE) comparing each of the true baseline hazard functions to the estimated baseline hazard functions are included

The operating characteristics of the regression estimates and the frailty parameter are displayed in Table 1. Both modeling approaches resulted in regression estimates with little bias and good coverage. For the Weibull-based model, the estimates of the frailty variance parameter were slightly underestimated with conservative coverage. For the B-spline based model, the mean of the frailty variance parameter was upward downward. The simulation results for the baseline hazard parameters corresponding to the fitted Weibull-based models are displayed in Additional file 1: Table B.1 and exhibit negligible bias and good coverage. In Fig. 2, we display the true baseline hazard functions in red and the estimated baseline hazard functions from the fitted B-spline-based models in the left column and the corresponding kernel smooth Nelson-Aalen estimates from separate univariate Cox models in the right column. Deviations in the estimated baseline hazard functions for both approaches are likely due to data sparsity in the latter half of the follow-up time. The median integrated squared error comparing each of the true baseline hazard functions to the estimated baseline hazard functions over the range of observed event times are displayed in Fig. 2 and indicate that the B-spline-based model provides estimates of the baseline hazard functions that are close to the truth and are comparable, if not slightly better, than the kernel smooth Nelson-Aalen estimates corresponding to separate Cox models.

**Table 1:** Simulation results

		Weibull-based model				B-spline-based model			
Parameter	Truth	Est.	SE _*a*_	SE _*e*_	Cover.	Est.	SE _*a*_	SE _*e*_	Cover.
log*θ*	-1.39	-1.56	0.27	0.27	0.99	-0.97	0.30	0.34	0.53
*β*_1_	-0.03	-0.02	0.06	0.06	0.95	-0.05	0.07	0.06	0.95
*β*_2_	-0.33	-0.33	0.04	0.04	0.95	-0.36	0.05	0.05	0.93
*β*_3_	-0.11	-0.10	0.08	0.08	0.95	-0.13	0.09	0.08	0.96

One thousand data sets were generated under a model with Weibull baseline hazard functions described in “Simulation study” section with *n*=5,000 and were fit to models with Weibull and B-spline parameterized baseline hazard functions. Point estimates and analytical standard errors (SE _*a*_) were averaged across estimates. Empirical standard errors (SE _*e*_) correspond to the standard deviation of the parameter sampling distributions. Coverage was calculated as the proportion of estimated 95% Wald-based confidence intervals that contained the true parameter

### Assessing the impact of education level on incident dementia in a large US cohort

A description of the semi-competing risks data and demographic variables used in the applied analysis are presented in Table 2. Of the 33,117 members in our analytic dataset, elementary or high school was the highest level of education in 43% of members, followed by trade school, some college or a college degree in 40%, and post-graduate study in 16.7%. More than half (56.6%) were female and 72.0% were of White race. By the end of the study, nearly half (47.1%) of members died without a diagnosis of dementia, with 19.1% censored before dementia or death, 5.0% alive with dementia, and 28.9% died carrying a diagnosis of dementia. In examining the outcome prevalences among the three education level groups, there were more dementia diagnoses and all-cause deaths among members in the elementary and high school group.

**Table 2:** Description of semi-competing risks data and demographic variables in the analytic cohort from Kaiser Permanente Northern California

	Total		Cens before dementia or death		Alive with dementia		Died without dementia		Died with dementia	
	n	(%)	n	(%)	n	(%)	n	(%)	n	(%)
Total	33,117	(100.0)	6,310	(19.1)	1,641	(5.0)	15,593	(47.1)	9,573	(28.9)
Education										
Elementary, High School	14,325	(43.3)	2,330	(16.3)	711	(5.0)	6,858	(47.9)	4,426	(30.9)
Trade degree, College	13,273	(40.1)	2,625	(19.8)	666	(5.0)	6,259	(47.2)	3,723	(28.0)
Graduate	5,519	(16.7)	1,355	(24.6)	264	(4.8)	2,476	(44.9)	1,424	(25.8)
Sex										
Female	18,748	(56.6)	3,880	(20.7)	1,152	(6.1)	7,918	(42.2)	5,798	(30.9)
Male	14,369	(43.4)	2,430	(16.9)	489	(3.4)	7,675	(53.4)	3,775	(26.3)
Race/ethnicity										
White	23,830	(72.0)	4,381	(18.4)	973	(4.1)	11,646	(48.9)	6,830	(28.7)
Black	5,172	(15.6)	881	(17.0)	355	(6.9)	2,237	(43.3)	1,699	(32.8)
Asian	2,327	(7.0)	630	(27.1)	178	(7.6)	981	(42.2)	538	(23.1)
Latinx	1,788	(5.4)	418	(23.4)	135	(7.6)	729	(40.8)	506	(28.3)

The regression estimates from the fitted models are presented in Table 3 and Table C.1 of the Additional file 1. Across all models and time origins, increasing education level was associated with a decreased risk of dementia. In the primary analysis (on the age scale with the inclusion of a shared frailty presented in the first two columns of Table 3), the estimated hazard ratio (HR) and 95% confidence interval (CI) was 0.87 (0.83, 0.92) comparing those with a trade degree, some college or a college degree to those who had an elementary or high school education. This protective effect was amplified when comparing those with post-graduate study to those who had an elementary or high school education (HR: 0.76, 95% CI: 0.71, 0.81). There was some variation in the estimated HR across different time origins and models. The hazard ratio comparing those with post-graduate study to those who had an elementary or high school education for the time-on-study model with shared frailty (third column of Table 3) was closer to the null with value 0.80 and to a greater degree in the time-on-study model without shared frailty (last two columns of Table 3) with an estimated HR of 0.83.

**Table 3:** Estimated regression parameters from analyses of Kaiser data based on an illness-death model with B-spline parameterized baseline hazard functions

	With shared frailty						Without shared frailty					
	Years since age 65			Years since study entry			Years since age 65			Years since study entry		
Parameter	HR	95% CI	*p*-value ^∗^	HR	95% CI	*p*-value ^∗^	HR	95% CI	*p*-value ^∗^	HR	95% CI	*p*-value ^∗^
Frailty variance, *θ*	0.41	(0.37,0.45)	<.001	0.25	(0.2,0.31)	<.001						
Dementia												
Trade degree, College	0.87	(0.83,0.92)	<.001	0.89	(0.85,0.94)	<.001	0.88	(0.84,0.92)	<.001	0.91	(0.87,0.95)	<.001
Post-graduate	0.76	(0.71,0.81)		0.80	(0.74,0.85)		0.81	(0.76,0.86)		0.83	(0.78,0.88)	
Female	1.01	(0.96,1.05)	0.40	1.02	(0.98,1.07)	0.17	1.00	(0.95,1.04)	0.44	1.03	(0.99,1.08)	0.06
Black	1.42	(1.34,1.51)	<.001	1.42	(1.34,1.51)	<.001	1.31	(1.24,1.38)	<.001	1.38	(1.31,1.46)	<.001
Asian	0.81	(0.74,0.89)		0.90	(0.82,0.98)		0.80	(0.73,0.87)		0.90	(0.83,0.98)	
Latinx	0.99	(0.89,1.09)		1.07	(0.97,1.17)		1.07	(0.98,1.16)		1.06	(0.97,1.16)	
Age at study entry				2.03	(1.98,2.08)	<.001				1.97	(1.93,2.02)	<.001
Death												
Trade degree, College	0.84	(0.81,0.88)	<.001	0.85	(0.81,0.88)	<.001	0.84	(0.81,0.87)	<.001	0.88	(0.85,0.92)	<.001
Post-graduate	0.68	(0.65,0.72)		0.69	(0.65,0.73)		0.73	(0.69,0.77)		0.75	(0.71,0.79)	
Female	0.62	(0.60,0.65)	<.001	0.62	(0.60,0.64)	<.001	0.62	(0.60,0.65)	<.001	0.64	(0.62,0.67)	<.001
Black	1.02	(0.96,1.07)	<.001	1.05	(0.99,1.11)	<.001	1.03	(0.98,1.08)	<.001	1.01	(0.96,1.06)	<.001
Asian	0.70	(0.65,0.76)		0.77	(0.71,0.83)		0.81	(0.76,0.87)		0.76	(0.70,0.82)	
Latinx	0.72	(0.65,0.79)		0.78	(0.71,0.85)		0.76	(0.70,0.82)		0.77	(0.71,0.84)	
Age at study entry				1.62	(1.59,1.66)	<.001				1.57	(1.55,1.60)	<.001
Death following dementia												
Trade degree, College	0.90	(0.85,0.96)	<.001	1.02	(0.96,1.09)	0.52	0.99	(0.93,1.05)	0.001	1.03	(0.97,1.10)	0.23
Post-graduate	0.77	(0.71,0.84)		1.05	(0.96,1.15)		0.86	(0.79,0.93)		1.07	(0.99,1.16)	
Female	0.67	(0.63,0.70)	<.001	1.02	(0.95,1.08)	0.31	0.68	(0.64,0.71)	<.001	1.01	(0.95,1.07)	0.37
Black	0.86	(0.79,0.92)	<.001	1.02	(0.94,1.10)	0.19	0.95	(0.88,1.02)	<.001	0.99	(0.92,1.06)	0.90
Asian	0.69	(0.60,0.78)		1.09	(0.97,1.24)		0.69	(0.61,0.78)		1.01	(0.90,1.13)	
Latinx	0.74	(0.64,0.84)		1.08	(0.95,1.23)		0.90	(0.80,1.02)		1.00	(0.89,1.13)	
Age at dementia				1.01	(0.98,1.03)	0.31				1.00	(0.98,1.03)	0.37

Models were fit with and without a shared frailty term. Two time origins were considered: 1) age 65 (age scale, left-truncated data); and 2) study entry (time-on-study)

^*^p-value for a categorical variable with *k*>2 levels (educational attainment, race/ethnicity) is based on a (*k*−1)-degree of freedom Wald test of linear hypotheses

Similarly, across all models and time origins, an education level of some college or more compared with a trade degree or less was associated with a decreased risk of death, with estimated HR and 95% CI in the primary model of 0.84 (0.81, 0.88) comparing those with a trade degree, some college or a college degree to those who had an elementary or high school education and 0.68 (0.65, 0.72) comparing those with post-graduate education to those who had an elementary or high school education. In our primary model, increased education level was associated with a reduced risk of death following dementia diagnosis. However, the time-on-study models (columns 3, 4, 7 and 8 of Table 3) provide no evidence of this association with point estimates that suggest an opposite effect.

**Fig. 3:**
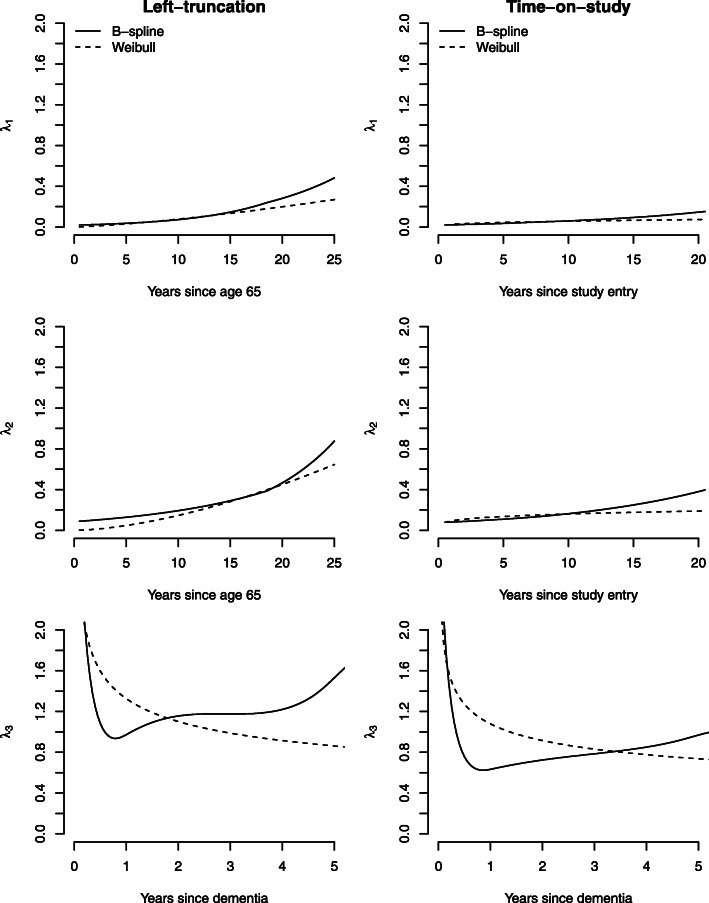
Estimated baseline hazard functions from analysis of Kaiser data assuming either B-spline or Weibull parameterized baseline hazard functions and shared frailty term for two time origins: 1) age 65 (age-scale, adjusted for left-truncation), and 2) study entry (time-on-study)

In the models with a shared frailty and B-spline baseline hazard functions (first four columns of Table 3), the estimated frailty variance parameter, *θ* was 0.41 (0.37, 0.45) when age was the time scale and 0.25 (0.20, 0.31) for the time-on-study analysis, suggesting a greater degree of dependence between time-to-dementia and all-cause death when age is the time scale. When Weibull baseline hazards were assumed (presented in Table C.1 of the Additional file 1), the estimated frailty variance parameter, *θ* was comparable with value 0.36 (0.32, 0.40) when age was the time scale. However, the estimated frailty variance was null for the time-on-study analysis. This finding is difficult to explain, but an examination of the estimated baseline hazard functions from the frailty models indicate differences in the underlying baseline risk assumed in the various models. Figure 3 presents the estimated baseline hazard functions under B-spline or Weibull parameterizations and a shared frailty term for two time origins: 1) age 65 (age-scale, adjusted for left-truncation), and 2) study entry (time-on-study). After fitting a suite of models that varied the number of internal knots and the B-spline polynomial degree, the specification with the largest log-likelihood was defined by one internal knot with linear B-splines for the 1- and 2-transitions and two internals knots with cubic B-splines for the 3-transition. We observe that the baseline hazard for the 1- and 2-transitions were similar for the B-spline and Weibull specifications and increasing over time, more so when time was on the age scale (left panel) than time-on-study (right panel). For the 3-transition (death following dementia), the estimated baseline hazard functions were quite different for the B-spline and Weibull parameterizations, as the Weibull can only accommodate baseline hazards of the form of a power function, while B-splines can approximate a range of functional forms. For both B-spline and Weibull parameterizations, the estimated baseline hazard for the 3-transition indicate that the risk of death is highest shortly after dementia diagnosis, which has been observed in a study of survival after dementia diagnosis in five racial/ethnic groups [45]. This is followed by either decreases then increases (based on the B-spline parameterization) or decreases over time (based on the Weibull parameterization).

## Discussion

For the analysis of left-truncated semi-competing risks data, we have provided methods and software for fitting an illness-death model with shared frailty assuming Weibull or B-spline baseline hazards. These methods were used to estimate the association of education level and dementia accounting for the competing risk of death in a cohort of 33,117 Kaiser Permanente Northern California members. We found a dose-response relationship between educational attainment and incident dementia, with a decreased risk of dementia associated with increasing levels of education, after adjusting for sex and race/ethnicity. The impact of education level and incident dementia is still not well understood, with published studies reporting both protective and null effects [46, 47]. Our study supports that higher education is associated with a lower risk of dementia in a large US cohort.

Note that the conclusions drawn for the outcome of interest (dementia) from the illness-death model with shared frailty aligned with those from alternative models that we considered, which omitted the shared frailty or used study entry as the time origin (as shown in Table 3). We expect that there will be variation in the estimates from these models depending on the data at hand, as was observed in the estimates of education level on the risk of death following dementia diagnosis in our study.

In our examination of model fitting operating characteristics via simulated data, we found that the regression parameters were estimated with negligible bias and good coverage. However, on average the frailty variance parameter was slightly underestimated for the Weibull baseline hazard parameterization and overestimated for the B-spline baseline hazard parameterization. For the case of the Weibull baseline hazard parameterization, the coverage was conservative for a sample size 5,000, but was closer to 95% when the sample size was increased 10,000 (see Table B.2 in the Additional file 1). For the case of the B-spline baseline hazard parameterization, the coverage was lower than 95% due to the bias in the frailty variance parameter. It is important to note that primary interest in the methods presented in this paper are the regression parameters. The frailty, and corresponding frailty variance parameter, allow us to further account for the dependence between the nonterminal and terminal events beyond covariate adjustment, analogous to a random effect in a random effects model. Similar to a random effects model, the primary interest lies in the mean outcome model and regression estimates; the variance parameter of the normally distributed random effects are typically of secondary interest.

In the analysis of dementia diagnoses such as those presented in this paper, prevalent cases at study entry are typically excluded. However, the likelihood in “Likelihood” section can be easily updated (see Additional file 1: Section D) to include prevalent nonterminal cases. Note that in the literature, there are two approaches for handling prevalent nonterminal cases in the analysis of left-truncated semi-competing risks data. The approach we take conditions on the history up to the left truncation time as in [25, 37] so that prevalent nonterminal cases only contribute to the estimation of *λ*_3_. Estimation is straight-forward assuming an illness-death model with shared frailty since the frailty term, *γ*, can be easily integrated out. Alternatively, Saarela and colleagues [24] provided methods for estimation that conditions on the left-truncation time only, so that prevalent cases contribute to the estimation of all transition hazards. This approach is more efficient as it uses more of the data, but is computationally more intensive as it involves numerical integration. This approach does not accommodate an illness-death model with shared frailty well since the integration of the shared frailty term is not straight-forward.

In our approach to fitting an illness-death model with shared frailty to left-truncated semi-competing risks data, we have considered fully-parametric specifications of the baseline hazard functions, Weibull and B-spline. Both functional forms are flexible and can approximate a wide range of baseline hazard functions. At the time of submission, a pre-print by Gorfine et al. [48] proposed a semi-parametric approach to the illness-death model with shared frailty using a pseudo-likelihood approach to estimating the regression parameters and baseline hazard functions that accommodates left-truncated semi-competing risks data. While the illness-death model with shared frailty in this paper was formulated using hazard models that are conditional on the frailty in (4)-(6), Gorfine et al. [48] focused on marginal Cox hazard models. This is analogous to the conditional and marginal approaches to modeling mean outcomes in the presence of clustering via mixed models [49] and generalized estimating equations [50], respectively. Thus our approaches are complementary, filling a gap in the literature and allowing the analyst options for fitting an illness-death model with shared frailty to left-truncated semi-competing risks data.

One of the reviewers pointed out that dementia diagnoses may be subject to interval-censoring. To explore the possibility and/or extent of interval-censoring in the cohort, we looked at the patterns of inpatient and outpatient visits (during which dementia might be assessed) among two groups of members: those who were diagnosed with dementia during the study, and those who died without a dementia diagnosis. The concern is that long gaps between visits would lead to imprecise dementia diagnosis dates in the former and missed opportunities for dementia diagnoses in the latter. We found that among those who were diagnosed with dementia during the study, 81% had a visit with a physician within 60 days prior to the diagnosis date in the EMR. For those who died without a dementia diagnosis, 90% had a visit within 60 days prior to death. Plots of individual-level visit patterns over the study period (see Supplementary File Figures C1 and C2) among members of these two groups illustrate that utilization is high in this cohort.

Based on these data, we believe that interval-censoring is not of major concern in our data, as it is in prospective studies of Alzheimer’s disease or dementia, such as PAQUID [51, 52] and the Adult Changes in Thought Study [53, 54], where dementia screening can be years apart. While analyses of data from those prospective studies are indeed complicated by interval-censoring, they were designed for the purpose of understanding incident dementia with identification of dementia cases based on a battery of neuropsychological testing and confirmation by a neurologist. An EMR-based analysis of a cohort of high care utilizers may avoid interval-censoring, however, may capture dementia cases with less rigor. At KPNC, a similar set of EMR code used to identify dementia diagnoses was shown to have a sensitivity of 77% and a specificity of 95% compared with a consensus dementia diagnosis utilizing a neuropsychiatric battery, structured interviews, physical examination, and medical records review. If interval-censoring were evident in our data, modeling should be updated to account for interval-censoring. This can be done by updating the likelihood function, as in Touraine, et al. 2017 [52]. In the setting of a shared frailty illness-death model presented in this paper, this is an avenue for future research.

It is important to mention that we provide methods for fitting a shared frailty illness-death model subject to left-truncated data when the covariate of interest is fixed with time. As one reviewer aptly pointed out, time-varying covariates may also be of interest to an analyst. We believe that the modeling framework specified in this paper can incorporate time-dependent covariates. However, deriving the likelihood function for a shared frailty model requires marginalization (integration) of the frailty term, which is complicated when time-dependent covariates are used and, such, beyond the scope of this paper. We intend to explore the implementation of the proposed extension with time-varying covariates in future work.

## Conclusions

As illustrated by our analysis of Kaiser data, our proposed modeling framework allows the analyst to assess the impact of covariates on semi-competing risks data, such as incident dementia and death, while accounting for dependence between the outcomes when data are left-truncated, as is common in studies of aging and dementia. This approach has the potential to be applied to a wide range of settings beyond the field of aging.

## Supplementary Information

**Additional file 1** Supplementary findings. File name: Lee_Gilsanz_Haneuse-Additional file.pdf. Includes: A: Marginal components of the likelihood referenced in “Likelihood” section; B: Additional simulation results; C: Additional information from the analysis of Kaiser data including: detailed description of the data from Kaiser Permanente Northern California and additional results from the analysis of Kaiser data; D: Likelihood expression when observed data include prevalent nonterminal cases referenced in “Discussion” section.

Additional file r code for implementation. File names: fit-models.txt and functions.txt provide code for generating simulated semi-competing risks data and fitting the shared frailty illness-death model to left-truncated semi-competing risks data described in the manuscript.

## Data Availability

The data used in this study contain Protected Health Information of KPNC members and cannot be shared. Code for generating simulated data used in the paper and that were similar to those analyzed in the data application are provided as additional files.

## Abbreviations

KPNC: Kaiser Permanente Northern California
HR: Hazard ratio
CI: Confidence interval
