# Supplementary material for: Fitting a shared frailty illness-death model to left-truncated semi-competing risks data to examine the impact of education level on incident dementia
Source: BMC Med Res Methodol. 2021 Jan 11;21:18. doi: 10.1186/s12874-020-01203-8 (PMC7802231; doi:10.1186/s12874-020-01203-8)
Supplement: Supplementary file 1 — Additional file 1 Supplementary findings. File name: Lee_Gilsanz_Haneuse-Additional file.pdf. Includes: A: Marginal components of the likelihood referenced in “Likelihood” section; B: Additional simulation results; C: Additional information from the analysis of Kaiser data including: detailed description of the data from Kaiser Permanente Northern California and additional results from the analysis of Kaiser data; D: Likelihood expression when observed data include prevalent nonterminal cases referenced in “Discussion” section. [file 12874_2020_1203_MOESM1_ESM.pdf]

Additional File for

“Fitting a shared frailty illness-death model to left-truncated semi-competing risks data to examine the impact of education level on incident dementia"

Catherine Lee
Paola Gilsanz
Division of Research,
Kaiser Permanente, Oakland, California, U.S.A.
catherine.lee@kp.org

Sebastien Haneuse
Department of Biostatistics,
Harvard T. H. Chan School of Public Health, Boston, Massachusetts, U.S.A.

Introduction

We present additional results that are beyond what could be presented in the main manuscript. This document is organized as follows:

Section A: Components of marginal log-likelihood function referenced in Section 3 of the main manuscript

Section B: Additional simulation results

Section C: Additional information from the analysis of Kaiser data including:

C.1 Further details regarding data from Kaiser Permanente Northern California

C.2 Additional results from the analysis of Kaiser data

Section D: Likelihood function when prevalent nonterminal cases are included referenced in Section 6 of the main manuscript.

A Marginal likelihood components

The likelihood components presented in Section 3 of the main manuscript are conditional on the frailty terms, which are unobserved. Below we present the marginal likelihood components which are obtained by marginalizing (integrating out) the frailty terms, γ. We assumed the following parameterization of the Gamma distribution, $X \sim \Gamma(\alpha, \beta)$, with $E(X) = \alpha/\beta$ and density:

$$f(x|\alpha, \beta) = \frac{\beta^{\alpha}}{\Gamma(\alpha)} x^{\alpha-1} \exp\{-\beta x\},$$

so that $\gamma \sim \Gamma(\alpha = 1/\theta, \beta = 1/\theta)$ has mean 1 and corresponds to the following density:

$$f_{\gamma}(\gamma|\alpha = 1/\theta, \beta = 1/\theta) = \frac{\theta^{-\frac{1}{\theta}}}{\Gamma\left(\frac{1}{\theta}\right)} \gamma^{\frac{1}{\theta}-1} \exp\left\{-\frac{\gamma}{\theta}\right\}.$$

$$\begin{aligned}
f_{(1)}(y_1, y_2|x) &= \int_0^{\infty} f_{(1)}(y_1, y_2|\gamma, x) f_{\gamma}(\gamma|\theta)\ d\gamma \\
&= \int_0^{\infty} \gamma^2\ \lambda_1^*(y_1|x)\lambda_3^*(y_2|x) \exp\{-\gamma(k_1 + k_2)\} \frac{\theta^{-\frac{1}{\theta}}}{\Gamma\left(\frac{1}{\theta}\right)} \gamma^{\frac{1}{\theta}-1} \exp\left\{-\frac{\gamma}{\theta}\right\}\ d\gamma\ \cdot\ I(l < y_1) \\
&= \lambda_1^*(y_1|x)\lambda_3^*(y_2|x) \frac{\theta^{-\frac{1}{\theta}}}{\Gamma\left(\frac{1}{\theta}\right)} \int_0^{\infty} \gamma^{2+\frac{1}{\theta}-1}\ \exp\left\{-\gamma\left(k_1 + k_2 + \frac{1}{\theta}\right)\right\}\ d\gamma\ \cdot\ I(l < y_1), \\
&\text{where } k_1 = \int_0^{y_2 - y_1} \lambda_3^*(u|y_1, x)\ du \text{ and } k_2 = \int_l^{y_1} (\lambda_1^*(u|x) + \lambda_2^*(u|x))\ du. \\
&\text{Note the integrand is the kernel of a } \Gamma\left(\alpha = 2 + \frac{1}{\theta},\ \beta = k_1 + k_2 + \frac{1}{\theta}\right) \\
&\text{so that the integral is known.} \\
&= \lambda_1^*(y_1|x)\lambda_3^*(y_2|x)(\theta + 1)((k_1 + k_2)\theta + 1)^{-\frac{1}{\theta}-2}\ \cdot\ I(l < y_1)
\end{aligned}$$

$$\begin{aligned}
f_{(2)}(y_1, y_2|x) &= \int_0^{\infty} \gamma\ \lambda_2^*(y_1|x) \exp\{-\gamma k_2\} \frac{\theta^{-\frac{1}{\theta}}}{\Gamma\left(\frac{1}{\theta}\right)} \gamma^{\frac{1}{\theta}-1} \exp\left\{-\frac{\gamma}{\theta}\right\}\ d\gamma\ \cdot\ I(l < y_1) \\
&= \lambda_2^*(y_1|x) \frac{\theta^{-\frac{1}{\theta}}}{\Gamma\left(\frac{1}{\theta}\right)} \int_0^{\infty} \gamma^{1+\frac{1}{\theta}-1}\ \exp\left\{-\gamma\left(k_2 + \frac{1}{\theta}\right)\right\}\ d\gamma\ \cdot\ I(l < y_1) \\
&= \lambda_2^*(y_1|x)(k_2\theta + 1)^{-1-\frac{1}{\theta}} \cdot\ I(l < y_1)
\end{aligned}$$

$$\begin{aligned}
f_{(3)}(y_1, y_2|x) &= \int_0^{\infty} \gamma\ \lambda_1^*(y_1|x) \exp\{-\gamma(k_1 + k_2)\} \frac{\theta^{-\frac{1}{\theta}}}{\Gamma\left(\frac{1}{\theta}\right)} \gamma^{\frac{1}{\theta}-1} \exp\left\{-\frac{\gamma}{\theta}\right\}\ d\gamma\ \cdot\ I(l < y_1) \\
&= \lambda_1^*(y_1|x) \frac{\theta^{-\frac{1}{\theta}}}{\Gamma\left(\frac{1}{\theta}\right)} \int_0^{\infty} \gamma^{1+\frac{1}{\theta}-1}\ \exp\left\{-\gamma\left(k_1 + k_2 + \frac{1}{\theta}\right)\right\}\ d\gamma\ \cdot\ I(l < y_1) \\
&= \lambda_1^*(y_1|x)((k_1 + k_2)\theta + 1)^{-1-\frac{1}{\theta}} \cdot\ I(l < y_1)
\end{aligned}$$

$$\begin{aligned}
f_{(4)}(y_1, y_2|, x) &= \int_0^{\infty} \exp\{-\gamma k_2\} \frac{\theta^{-\frac{1}{\theta}}}{\Gamma\left(\frac{1}{\theta}\right)} \gamma^{\frac{1}{\theta}-1} \exp\left\{-\frac{\gamma}{\theta}\right\}\ d\gamma\ \cdot\ I(l < y_1) \\
&= (k_2\theta + 1)^{-\frac{1}{\theta}}\ \cdot\ I(l < y_1)
\end{aligned}$$

$$
\begin{aligned}
f_{(5)}(y_1, y_2|x) &= \int_0^\infty \gamma\ \lambda_3^*(y_2|y_1, x) \exp\{-\gamma k_3\} \frac{\theta^{-\frac{1}{\theta}}}{\Gamma\left(\frac{1}{\theta}\right)} \gamma^{\frac{1}{\theta}-1} \exp\left\{-\frac{\gamma}{\theta}\right\}\ d\gamma \ \cdot\ I(y_1 \leq l < y_2) \\
&= \lambda_3^*(y_2|y_1, x) \frac{\theta^{-\frac{1}{\theta}}}{\Gamma\left(\frac{1}{\theta}\right)} \int_0^\infty \gamma^{1+\frac{1}{\theta}-1}\ \exp\left\{-\gamma\left(k_3 + \frac{1}{\theta}\right)\right\}\ d\gamma \ \cdot\ I(y_1 \leq l < y_2) \\
&= \lambda_3^*(y_2|y_1, x)\,(k_3\theta + 1)^{-1-\frac{1}{\theta}} \cdot\ I(y_1 \leq l < y_2), \\
&\text{where } k_3 = \int_{l-y_1}^{y_2-y_1} \lambda_3^*(u|x)\ du.
\end{aligned}
$$

$$
\begin{aligned}
f_{(6)}(y_1, y_2|, x) &= \int_0^\infty \exp\{-\gamma k_3\} \frac{\theta^{-\frac{1}{\theta}}}{\Gamma\left(\frac{1}{\theta}\right)} \gamma^{\frac{1}{\theta}-1} \exp\left\{-\frac{\gamma}{\theta}\right\}\ d\gamma \ \cdot\ I(y_1 \leq l < y_2) \\
&= (k_3\theta + 1)^{-\frac{1}{\theta}} \quad \cdot \quad I(y_1 \leq l < y_2)
\end{aligned}
$$

B Additional simulation results

Table B.1: Full simulation results for the Weibull baseline hazard model. One thousand data sets were generated under a model with Weibull baseline hazard functions described in Section 4.1 of the main manuscript with $n = 5,000$ observations. Point estimates were obtained by averaging over all 1,000 estimated parameters. Analytical standard errors, SE_a, were averaged over 1,000 estimated standard error estimates based on the information matrix. Empirical standard errors, SE_e, correspond to the standard deviation of the parameter sampling distributions. Coverage was calculated as the proportion of estimated 95% Wald-based confidence intervals that contained the true parameter.

Parameter	Truth	Est.	SE_a	SE_e	Cover.
$\log(\kappa_1)$	-9.98	-9.88	0.30	0.31	0.93
$\log(\alpha_1)$	1.05	1.04	0.04	0.04	0.93
$\log(\kappa_2)$	-10.01	-9.91	0.26	0.26	0.94
$\log(\alpha_2)$	1.15	1.14	0.03	0.03	0.93
$\log(\kappa_3)$	-5.92	-5.92	0.18	0.18	0.94
$\log(\alpha_3)$	0.92	0.92	0.03	0.03	0.94
$\log(\theta)$	-1.39	-1.56	0.27	0.27	0.99
β_1	-0.03	-0.02	0.06	0.06	0.95
β_2	-0.33	-0.33	0.04	0.04	0.95
β_3	-0.11	-0.10	0.08	0.08	0.95

Table B.2: Full simulation results for the Weibull baseline hazard model. One thousand data sets were generated under a model with Weibull baseline hazard functions described in Section 4.1 of the main manuscript with $n = 10,000$ observations. Point estimates were obtained by averaging over all 1,000 estimated parameters. Analytical standard errors, SE_a, were averaged over 1,000 estimated standard error estimates based on the information matrix. Empirical standard errors, SE_e, correspond to the standard deviation of the parameter sampling distributions. Coverage was calculated as the proportion of estimated 95% Wald-based confidence intervals that contained the true parameter.

Parameter	Truth	Est.	SE_a	SE_e	Cover.
$\log(\kappa_1)$	-9.98	-9.88	0.22	0.22	0.92
$\log(\alpha_1)$	1.05	1.04	0.03	0.03	0.91
$\log(\kappa_2)$	-10.01	-9.90	0.18	0.19	0.90
$\log(\alpha_2)$	1.15	1.14	0.02	0.02	0.88
$\log(\kappa_3)$	-5.92	-5.92	0.13	0.13	0.96
$\log(\alpha_3)$	0.92	0.92	0.02	0.02	0.95
$\log(\theta)$	-1.39	-1.54	0.18	0.19	0.93
β_1	-0.03	-0.02	0.04	0.04	0.95
β_2	-0.33	-0.33	0.03	0.03	0.94
β_3	-0.11	-0.10	0.06	0.06	0.95

C Additional information from the analysis of Kaiser data

C.1 Detailed description of the data from Kaiser Permanente Northern California

Study population. Analyses follow members of Kaiser Permanente Northern California (KPNC) who participated in the Multiphasic Health Checkups (MHC) at least once between 1964-1996, and were at least 65 years old and KPNC members as of 1/1/1996. KPNC is an integrated healthcare delivery system. The member population is generally representative of the catchment area population, with the caveat that individuals at extreme tails of the income distribution are underrepresented **?**. MHC was an optional check-up provided to health plan members in San Francisco and Oakland, California. Our analyses includes the first visit of 36,134 members with information regarding their level of education, smoking, and hypertension status during the MHC and no dementia diagnosis prior to 1997.

Education. MHC questionnaires captured information on participant educational attainment. Participants were asked the highest grade they completed (response options: 0-6; 7-9; 10-11; 12; Technical/Business; Partial college; college graduate; post-graduate). If an individual completed additional education between 1964 and 1996, the highest level of educational attainment was utilized.

Dementia Diagnosis. Consistent with prior studies in this population **???**, dementia cases between January 1, 1997 and Sept 30, 2017 were identified using KPNC electronic medical records,. The following International Classification of Diseases, Ninth Revision (ICD-9) and Tenth Revision (ICD-10) diagnosis codes were used to identify dementia cases: vascular dementia (ICD-9: 290.4x; ICD-10: F01.5x), Alzheimer's disease (ICD-9: 331.0; ICD-10: G30.0, G30.1, G30.8, G30.9), and other/nonspecific dementia (ICD-9: 290.0, 290.1x, 290.2x, 290.3, 294.2x, and 294.8; ICD-10: F03.9x). A similar set of ICD-9 codes was shown to have a sensitivity of 77% and a specificity of 95% compared with a consensus dementia diagnosis utilizing a neuropsychiatric battery, structured interviews, physical examination, and medical records review **?**.

Covariates. Demographics obtained from KPNC records include age, race/ethnicity, and gender.

After excluding observations with missing covariate values and where the nonterminal and terminal event times coincided, the resulting dataset included 34,362 members. The data were then subset to those of White, Black, Asian and Latinx race/ethnicity resulting in a final analytic dataset of 33,117 members.

Figure C.1: Inpatient and outpatient visits for 100 randomly selected individuals who were diagnosed with dementia during the study.

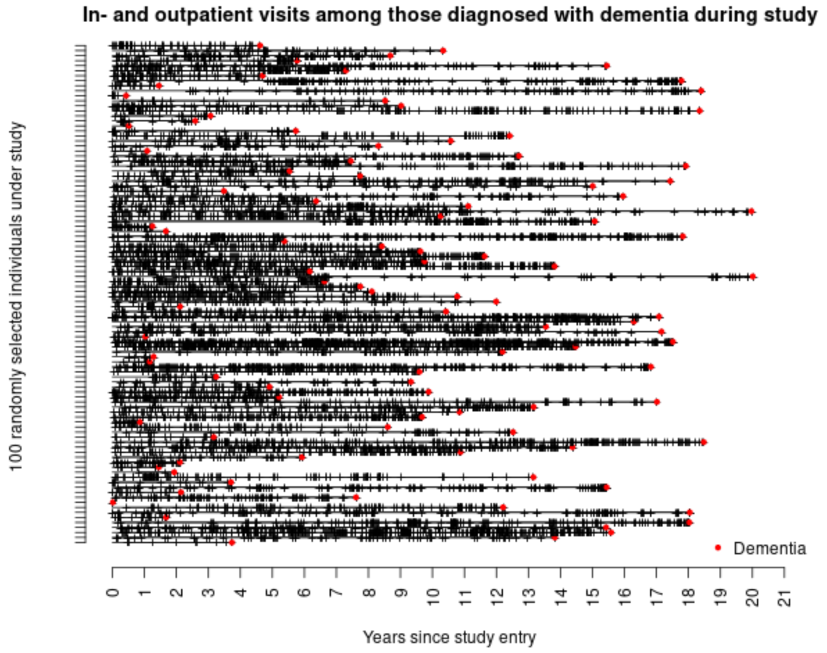

Figure C.2: Inpatient and outpatient visits for 100 randomly selected individuals who were died without a dementia diagnosis during the study.

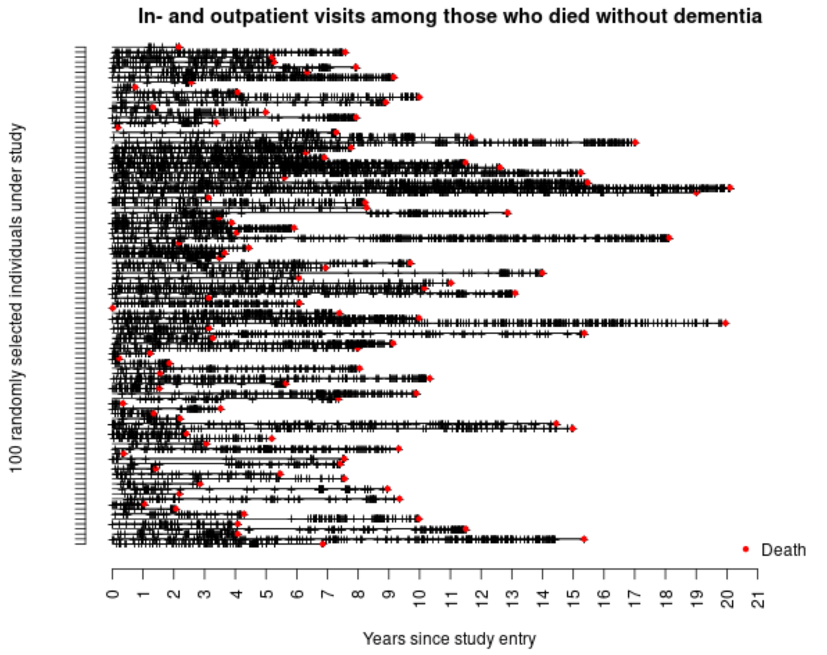

C.2 Additional results from the analysis of Kaiser data

Table C.1: Estimated regression parameters from analyses of Kaiser data based on an illness-death model with Weibull parameterized baseline hazard functions. Data were subset to individuals of white and black race/ethnicity. Models were fit with and without a shared frailty term. Two time origins were considered: 1) age 65 (left-truncated data); and 2) study entry.

	With shared frailty						Without shared frailty					
	Years since age 65			Years since study entry			Years since age 65			Years since study entry		
Parameter	HR	95% CI	p-value*	HR	95% CI	p-value*	HR	95% CI	p-value*	HR	95% CI	p-value*
Frailty variance, θ	0.36	(0.32,0.40)	<.001	0.00	(0,0)							
Dementia												
Trade degree, College	1.00	(0.95,1.05)	<.001	0.93	(0.85,1.01)	0.04	0.96	(0.92,1.00)	<.001	0.93	(0.85,1.01)	0.04
Post-graduate	0.87	(0.81,0.93)		0.86	(0.76,0.97)		0.88	(0.83,0.94)		0.86	(0.76,0.97)	
Female	1.15	(1.10,1.21)	<.001	1.04	(0.95,1.13)	0.41	1.08	(1.04,1.13)	<.001	1.04	(0.95,1.13)	0.41
Black	1.50	(1.42,1.60)	<.001	1.43	(1.29,1.60)	<.001	1.45	(1.37,1.53)	<.001	1.43	(1.29,1.60)	<.001
Asian	0.77	(0.71,0.85)		0.87	(0.73,1.03)		0.94	(0.87,1.02)		0.87	(0.73,1.03)	
Latinx	0.87	(0.79,0.97)		1.06	(0.88,1.26)		1.17	(1.07,1.27)		1.06	(0.88,1.26)	
Age at study entry				1.86	(1.78,1.94)	<.001				1.86	(1.78,1.94)	<.001
Death												
Trade degree, College	0.90	(0.86,0.94)	<.001	0.89	(0.82,0.96)	<.001	0.88	(0.84,0.91)	<.001	0.89	(0.82,0.96)	<.001
Post-graduate	0.73	(0.68,0.77)		0.77	(0.69,0.85)		0.78	(0.74,0.83)		0.77	(0.69,0.85)	
Female	0.67	(0.64,0.69)	<.001	0.64	(0.60,0.69)	<.001	0.64	(0.62,0.67)	<.001	0.64	(0.60,0.69)	<.001
Black	1.11	(1.05,1.17)	<.001	1.02	(0.92,1.13)	<.001	1.10	(1.04,1.16)	<.001	1.02	(0.92,1.13)	<.001
Asian	0.66	(0.61,0.72)		0.73	(0.63,0.85)		0.78	(0.72,0.84)		0.73	(0.63,0.85)	
Latinx	0.75	(0.68,0.82)		0.78	(0.66,0.93)		0.85	(0.79,0.93)		0.78	(0.66,0.93)	
Age at study entry				1.49	(1.43,1.54)	<.001				1.49	(1.43,1.54)	<.001
Death following dementia												
Trade degree, College	0.85	(0.80,0.91)	<.001	1.05	(0.93,1.18)	0.58	0.94	(0.88,1.00)	<.001	1.05	(0.93,1.18)	0.58
Post-graduate	0.77	(0.70,0.84)		0.97	(0.82,1.15)		0.81	(0.74,0.88)		0.97	(0.82,1.15)	
Female	0.68	(0.64,0.72)	<.001	0.94	(0.84,1.05)	0.27	0.71	(0.67,0.75)	<.001	0.94	(0.84,1.05)	0.27
Black	0.83	(0.76,0.90)	<.001	0.98	(0.85,1.14)	0.26	0.88	(0.82,0.95)	<.001	0.98	(0.85,1.14)	0.26
Asian	0.93	(0.82,1.05)		0.84	(0.67,1.06)		0.73	(0.65,0.83)		0.84	(0.67,1.06)	
Latino	0.78	(0.68,0.90)		0.89	(0.69,1.14)		0.80	(0.71,0.91)		0.89	(0.69,1.15)	
Age at dementia				1.01	(0.96,1.06)	0.66				1.01	(0.96,1.06)	0.66

* p-value for a categorical variable with $k > 2$ levels (educational attainment, race/ethnicity) is based on a $(k-1)$-degree of freedom Wald test of linear hypotheses.

Figure C.3: Estimated baseline hazard functions from analysis of KHANDLE DATA assuming B-spline baseline hazard functions and NO shared frailty term for two time origins: 1) age 65 (adjusted for left-truncation), and 2) study entry.

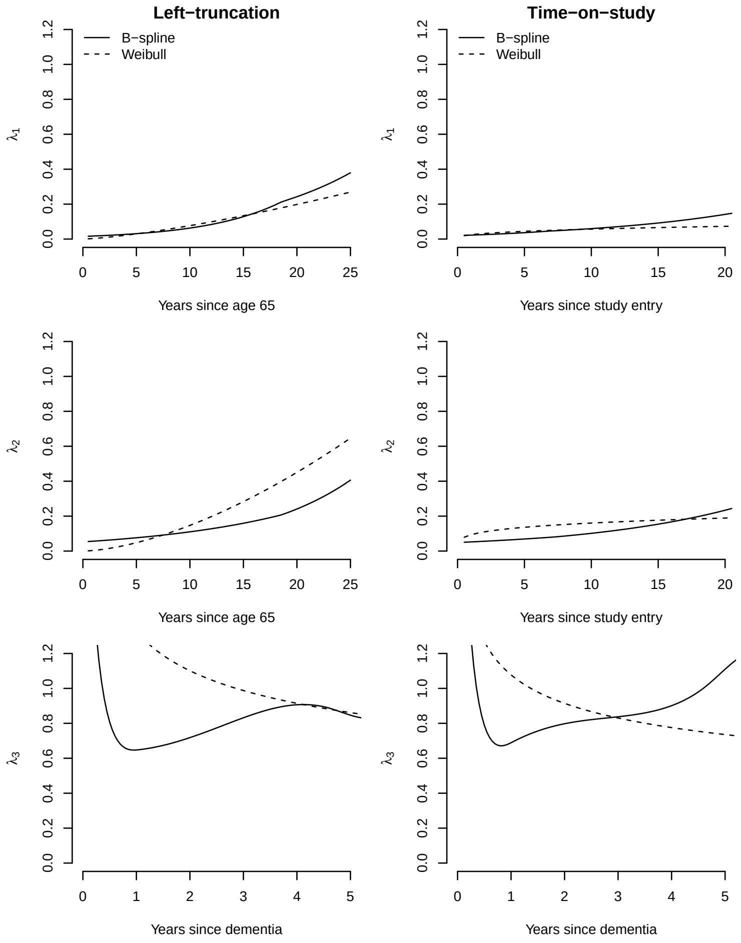

D Likelihood function when prevalent nonterminal cases are included

There are six possible likelihood contributions:

Table D.1: Likelihood contributions

Description	Observed data
(1) Healthy at sampling, both nonterminal and terminal events observed	$L_i < Y_{1i},\ \delta_1 = 1,\ \delta_2 = 1$
(2) Healthy at sampling, terminal event observed without nonterminal event	$L_i < Y_{1i},\ \delta_1 = 0,\ \delta_2 = 1$
(3) Healthy at sampling, both nonterminal observed, censored before terminal event	$L_i < Y_{1i},\ \delta_1 = 1,\ \delta_2 = 0$
(4) Healthy at sampling, censored before either event	$L_i < Y_{1i},\ \delta_1 = 0,\ \delta_2 = 0$
If $T_{1i} \leq L_i$ is observed:	
(5) Nonterminal event occurs before sampling, terminal event observed	$Y_{1i} \leq L_i < Y_{2i},\ \delta_1 = 1,\ \delta_2 = 1$
(6) Nonterminal event occurs before sampling, censored before terminal event	$Y_{1i} \leq L_i < Y_{2i},\ \delta_1 = 1,\ \delta_2 = 0$

$$
\begin{aligned}
&f_{(1)}(y_1, y_2|\gamma, x) \\
&= \frac{\lambda_1(y_1|\gamma,x)\lambda_3(y_2|\gamma,y_1,x)S_1(y_1|\gamma,x)S_2(y_1|\gamma,x)S_3(y_2-y_1|\gamma,y_1,x)}{S_1(l|\gamma,x)S_2(l|\gamma,x)} I(l < y_1) \\
&= \lambda_1(y_1|\gamma,x)\lambda_3(y_2|\gamma,y_1,x)\exp\left\{-\int_0^{y_2-y_1}\lambda_3(u|\gamma,y_1,x)\ du\right\}\exp\left\{-\int_l^{y_1}\left[\lambda_1(u|\gamma,x)+\lambda_2(u|\gamma,x)\right]\ du\right\} \\
&= \gamma^2\ \lambda_1^*(y_1|x)\lambda_3^*(y_2|x)\exp\left\{-\gamma\left[\int_0^{y_2-y_1}\lambda_3^*(u|y_1,x)\ du + \int_l^{y_1}\left(\lambda_1^*(u|x)+\lambda_2^*(u|x)\right)\ du\right]\right\} \\
&= \gamma^2\ \lambda_1^*(y_1|x)\lambda_3^*(y_2|x)\exp\left\{-\gamma\left(k_1+k_2\right)\right\}, \\
&\quad \text{where } k_1 = \int_0^{y_2-y_1}\lambda_3^*(u|y_1,x)\ du \text{ and } k_2 = \int_l^{y_1}\left(\lambda_1^*(u|x)+\lambda_2^*(u|x)\right)\ du.
\end{aligned}
$$

$$
\begin{aligned}
&f_{(2)}(y_1, y_2|\gamma, x) \\
&= \frac{\lambda_2(y_1|\gamma,x)S_1(y_1|\gamma,x)S_2(y_1|\gamma,x)}{S_1(l|\gamma,x)S_2(l|\gamma,x)} I(l < y_1) \text{ (Note: } y_1 = y_2 \text{ for this case.)} \\
&= \lambda_2(y_1|\gamma,x)\exp\left\{-\int_l^{y_1}\left[\lambda_1(u|\gamma,x)+\lambda_2(u|\gamma,x)\right]\ du\right\} \\
&= \gamma\ \lambda_2^*(y_1|x)\exp\left\{-\gamma\left[\int_l^{y_1}\left(\lambda_1^*(u|x)+\lambda_2^*(u|x)\right)\ du\right]\right\} \\
&= \gamma\ \lambda_2^*(y_1|x)\exp\left\{-\gamma k_2\right\}
\end{aligned}
$$

$$
\begin{aligned}
&f_{(3)}(y_1, y_2|\gamma, x) \\
&= \frac{\lambda_1(y_1|\gamma,x)S_1(y_1|\gamma,x)S_2(y_1|\gamma,x)S_3(y_2-y_1|\gamma,y_1,x)}{S_1(l|\gamma,x)S_2(l|\gamma,x)} I(l < y_1) \\
&= \lambda_1(y_1|\gamma,x)\exp\left\{-\int_0^{y_2-y_1}\lambda_3(u|\gamma,y_1,x)\ du\right\}\exp\left\{-\int_l^{y_1}\left[\lambda_1(u|\gamma,x)+\lambda_2(u|\gamma,x)\right]\ du\right\} \\
&= \gamma\ \lambda_1^*(y_1|x)\exp\left\{-\gamma\left[\int_0^{y_2-y_1}\lambda_3^*(u|y_1,x)\ du + \int_l^{y_1}\left(\lambda_1^*(u|x)+\lambda_2^*(u|x)\right)\ du\right]\right\} \\
&= \gamma\ \lambda_1^*(y_1|x)\exp\left\{-\gamma\left(k_1+k_2\right)\right\}
\end{aligned}
$$

$$
\begin{aligned}
&f_{(4)}(y_1, y_2|\gamma, x) \\
&= \frac{S_1(y_1|\gamma,x)S_2(y_1|\gamma,x)}{S_1(l|\gamma,x)S_2(l|\gamma,x)} I(l < y_1) \text{ (Note: } y_1 = y_2 \text{ for this case.)} \\
&= \exp\left\{-\int_l^{y_1}\left[\lambda_1(u|\gamma,x)+\lambda_2(u|\gamma,x)\right]\ du\right\} = \exp\left\{-\gamma\left[\int_l^{y_1}\left(\lambda_1^*(u|x)+\lambda_2^*(u|x)\right)\ du\right]\right\} = \exp\left\{-\gamma k_2\right\}
\end{aligned}
$$

$$
\begin{aligned}
&f_{(5)}(y_1, y_2|\gamma, x) \\
&= \frac{\lambda_1(y_1|\gamma,x)\lambda_3(y_2|\gamma,y_1,x)S_1(y_1|\gamma,x)S_2(y_1|\gamma,x)S_3(y_2-y_1|\gamma,y_1,x)}{\lambda_1(y_1|\gamma,x)S_1(y_1|\gamma,x)S_2(y_1|\gamma,x)S_3(l-y_1|\gamma,y_1,x)} I(y_1 \le l < y_2) \\
&= \frac{\lambda_3(y_2|\gamma,y_1,x)S_3(y_2-y_1|\gamma,y_1,x)}{S_3(l-t_1|t_1,z)} I(y_1 \le l < y_2) \\
&= \lambda_3(y_2|\gamma,y_1,x)\exp\left\{-\int_{l-y_1}^{y_2-y_1}\lambda_3(u|\gamma,x)\ du\right\} = \gamma\ \lambda_3^*(y_2|y_1,x)\exp\left\{-\gamma\int_{l-y_1}^{y_2-y_1}\lambda_3^*(u|x)\ du\right\} \\
&= \gamma\ \lambda_3^*(y_2|y_1,x)\exp\left\{-\gamma k_3\right\}, \\
&\quad \text{where } k_3 = \int_{l-y_1}^{y_2-y_1}\lambda_3^*(u|x)\ du.
\end{aligned}
$$

$$
\begin{aligned}
& f_{(6)}(y_1, y_2|\gamma, x) \\
&= \frac{\lambda_1(y_1|\gamma, x)S_1(y_1|\gamma, x)S_2(y_1|\gamma, x)S_3(y_2 - y_1|\gamma, y_1, x)}{\lambda_1(y_1|\gamma, x)S_1(y_1|\gamma, x)S_2(y_1|\gamma, x)S_3(l - y_1|\gamma, y_1, x)} I(y_1 \leq l < y_2) \\
&= \frac{S_3(y_2 - y_1|\gamma, y_1, x)}{S_3(l - t_1|t_1, z)} I(y_1 \leq l < y_2) \\
&= \exp\left\{-\int_{l-y_1}^{y_2-y_1} \lambda_3(u|\gamma, x)\ du\right\} = \exp\left\{-\gamma \int_{l-y_1}^{y_2-y_1} \lambda_3^*(u|x)\ du\right\} \\
&= \exp\{-\gamma k_3\}
\end{aligned}
$$
